# Azvudine for the Treatment of COVID‐19 in Pre‐Existing Cardiovascular Diseases: A Single‐Center, Real‐World Experience

**DOI:** 10.1002/advs.202306050

**Published:** 2024-03-27

**Authors:** Liu Wu, Zhong‐Han He, Ling Huang, Xin Guo, Xu‐Yong Li, Hong‐Da Zhang, Man‐Hua Chen

**Affiliations:** ^1^ Department of Cardiology, The Central Hospital of Wuhan, Tongji Medical College Huazhong University of Science and Technology Wuhan 430014 China; ^2^ State Key Laboratory of Cardiovascular Disease, Fuwai Hospital, National Center for Cardiovascular Diseases Chinese Academy of Medical Sciences & Peking Union Medical College Beijing 100037 China

**Keywords:** Azvudine, cardiovascular diseases, COVID‐19, death, outcome

## Abstract

COVID‐19 can lead to adverse outcomes in patients with pre‐existing diseases. Azvudine has been approved for treating COVID‐19 in China, but the real‐world data is limited. It is aimed to investigate the efficacy of Azvudine in patients with COVID‐19 and pre‐existing cardiovascular diseases. Patients with confirmed COVID‐19 and pre‐existing cardiovascular diseases are retrospectively enrolled. The primary outcome is all‐cause death during hospitalization. Overall, 351 patients are included, with a median age of 74 years, and 44% are female. 212 (60.6%) patients are severe cases. Azvudine is used in 106 (30.2%) patients and not in 245 (69.8%). 72 patients died during hospitalization. After multivariate adjustment, patients who received Azvudine a lower risk of all‐cause death (hazard ratio: 0.431; 95% confidence interval: 0.252–0.738; *p* = 0.002) than controls. Azvudine therapy is also associated with lower risks of shock and acute kidney injury. For sensitivity analysis in the propensity score‐matched cohort (*n* = 90 for each group), there is also a significant difference in all‐cause death between the two groups (hazard ratio: 0.189; 95% confidence interval: 0.071–0.498; *p* < 0.001). This study indicated that Azvudine therapy is associated with better outcomes in COVID‐19 patients with pre‐existing cardiovascular diseases.

## Graphical abstract

Azvudine has been approved for treating COVID‐19 in China, but the real‐world data is limited. This study shows that Azvudine therapy is associated with a lower risk of death in COVID‐19 patients with pre‐existing cardiovascular diseases. Azvudine therapy is also associated with lower risks of shock and acute kidney injury in these patients.

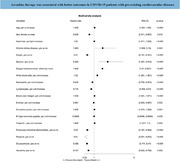

## 1 Introduction

The global Coronavirus Disease 2019 (COVID‐19) pandemic caused by the severe acute respiratory syndrome coronavirus 2 (SARS‐CoV‐2) has lasted over four years since the first case was identified in December 2019.^[^ ^1^ ^]^ Although the World Health Organization has declared that COVID‐19 is no longer a Public Health Emergency of International Concern, the pandemic itself is still ongoing and far from over.^[^ ^2^, ^3^, ^4^ ^]^ In China, we experienced a nationwide COVID‐19 surge dominated by the Omicron variant in the winter of 2022, after which we dropped quarantine measures against people infected with SARS‐CoV‐2 and stopped identifying close contacts or designating high‐risk and low‐risk areas.^[^ ^5^ ^]^ The 2022 winter surge was followed by several months of calm. However, we have been experiencing several waves of infections in the past year, although most patients are non‐severe cases, as we see in our clinical practice. The Chinese Center for Disease Control and Prevention has reported that the following surges were sporadic across the country, with fewer clinic visits and fewer deaths.^[^ ^6^ ^]^ However, the virus is still evolving and causing deaths worldwide.^[^ ^4^ ^]^ The possibility of new variants remains, potentially causing additional surges in future winters.

In China, Azvudine was officially approved as an alternative antiviral therapy for patients with COVID‐19 in 2022.^[^ ^7^ ^]^ Studies have shown that Azvudine could shorten the time for nucleic acid‐negative conversion and the symptom improvement time.^[^ ^8^, ^9^ ^]^ The efficacy of Azvudine on clinical outcomes, including death in general patients with COVID‐19, has been proved.^[^ ^10^ ^]^ However, a recent study indicated Azvudine did not reduce mortality in hospitalized COVID‐19 patients with pre‐existing conditions.^[^ ^11^ ^]^ Studies suggest that patients with pre‐existing conditions such as chronic obstructive pulmonary disease, diabetes, obesity, heart failure, and cancer are at increased risk of worse outcomes, including hospitalization, intensive care unit (ICU) admission, and mortality when infected with SARS‐CoV‐2. COVID‐19 patients with pre‐existing conditions require specialized attention and tailored treatment options.

In the present study, we aimed to investigate the efficacy of Azvudine in a unique group of COVID‐19 patients with pre‐existing cardiovascular diseases in a large tertiary center in Wuhan, China. This is a real‐world experience of Azvudine therapy during the nationwide COVID‐19 surge in the 2022 winter in China.

## 2 Results

### 2.1 Baseline Characteristics of the Overall Cohort

**Table** **1** displays the baseline characteristics and treatment information during the hospitalization of all patients. Overall, 351 patients with confirmed COVID‐19 and pre‐existing cardiovascular diseases were included in this study, with a median age of 74 years, and 44% were female. Of all patients, 212 (60.6%) were classified as severe cases (based on diagnosis at discharge). Azvudine was used in 106 (30.2%) patients and not in 245 (69.8%) patients. The two groups shared comparable demographic features and comorbidities (except for cardiomyopathy). There were more severe cases in patients who received Azvudine than those who did not (*p* < 0.001). More patients in the Azvudine group had respiratory symptoms and abnormal radiological findings than those in the non‐Azvudine group. The two groups of patients also differed in several laboratory parameters, including neutrophils, lymphocytes, albumin, and C‐reactive protein.

**Table 1:** Characteristics of patients in the overall cohort.

	All patients (*n* = 351)	Non‐Azvudine Group (*n* = 245)	Azvudine Group (*n* = 106)	*p* value^*^	Standardized mean difference
Age, yr	74 (16)	73 (17)	75 (18)	0.223	0.13
Sex female, *n* (%)	154 (44)	109 (45)	45 (43)	0.724	0.04
BMI, kg m^−2^	23.8 (4.2)	23.9 (4.5)	23.9 (4.2)	0.235	0.13
Systolic blood pressure, mmHg	128 (23)	128 (24)	130 (23)	0.823	0.05
Heart rate, bpm	82 (23)	80 (23)	87 (20)	0.009	0.23
SpO_2_, %	97 (3)	97 (3)	97 (6)	0.057	0.26
Severe COVID‐19, *n* (%)	212 (60.4)	131 (53.5)	81 (76.4)	< 0.001	0.50
Current smoker, *n* (%)	43 (12)	21 (11)	16 (15)	0.285	0.12
Comorbidities, *n* (%)					
Hypertension	223 (64)	150 (61)	73 (69)	0.172	0.16
Coronary artery disease	144 (41)	97 (40)	47 (44)	0.406	0.10
Heart failure	41 (12)	26 (11)	15 (14)	0.343	0.11
Arrhythmia	44 (13)	31 (13)	13 (12)	0.920	0.01
Cardiomyopathy	2 (0.6)	0 (0)	2 (2)	0.031	0.20
Valvular disease	5 (1.4)	4 (1.6)	1 (0.9)	0.617	0.06
Diabetes mellitus	113 (32)	76 (31)	37 (35)	0.474	0.08
Chronic lung disease	54 (15)	38 (16)	16 (15)	0.921	0.01
Chronic kidney disease	38 (11)	29 (12)	9 (9)	0.354	0.11
Chronic liver disease	2 (0.6)	2 (0.8)	0 (0)	0.351	0.13
Stroke or transient ischemic attack	36 (10)	25 (10)	11 (10)	0.961	0.01
Cancer	6 (1.7)	4 (1.6)	2 (1.9)	0.866	0.02
Immunosuppression	3 (0.9)	2 (0.8)	1 (0.9)	0.905	0.01
Other chronic diseases	28 (8)	16 (7)	12 (11)	0.128	0.17
Symptoms at admission, *n* (%)					
Fever	152 (43.3)	93 (38)	59 (56)	0.002	0.36
Cough	241 (68.7)	156 (64)	85 (80)	0.002	0.37
Sputum production	227 (64.7)	142 (58)	85 (80)	< 0.001	0.50
Shortness of breath	190 (54.1)	123 (50)	67 (63)	0.025	0.26
Diarrhea	4 (1.1)	4 (1.6)	0 (0)	0.186	0.18
Laboratory parameters					
White blood cells, 10^9^ L^−1^	6.1 (3.1)	6.7 (2.7)	6.3 (3.8)	0.187	0.13
Neutrophils, 10^9^ L^−1^	4.4 (3.1)	4.3 (2.9)	4.7 (3.9)	0.035	0.20
Lymphocytes, 10^9^ L^−1^	0.9 (0.6)	0.9 (0.6)	0.7 (0.6)	0.003	0.31
Hemoglobin, g L^−1^	125 (27)	125 (27)	126 (25)	0.946	0.01
Platelets, 10^9^ L^−1^	187 (115)	187 (111)	191 (121)	0.788	0.08
Serum creatinine, µ;mol L^−1^	76 (43)	76 (44)	75 (39)	0.501	0.05
Blood urea nitrogen, mg dl^−1^	6.3 (5.0)	6.3 (5.0)	6.5 (5.1)	0.316	0.02
Alanine aminotransferase, U L^−1^	22 (19)	22 (172)	23 (22)	0.156	0.02
Aspartate aminotransferase, U L^−1^	33 (28)	31 (25)	37 (29)	0.035	0.08
Albumin, g L^−1^	36 (7)	36 (8)	34 (4)	< 0.001	0.49
Total bilirubin, µ;mol L^−1^	12 (8)	12 (7)	12 (10)	0.886	0.03
Direct bilirubin, µ;mol L^−1^	4 (3)	4 (3)	5 (4)	0.235	0.08
Lactate dehydrogenase, U L^−1^	241 (139)	233 (122)	260 (145)	0.001	0.17
D‐dimer, ug mL^−1^	0.8 (1.1)	0.75 (1.03)	0.93 (1.38)	0.023	0.09
C‐reactive protein, mg L^−1^	4.3 (6.6)	4.3 (6.7)	5.5 (8.2)	< 0.001	0.31
Glycosylated hemoglobin, %	6.1 (0.7)	6.1 (0.7)	6.1 (0.7)	0.293	0.09
Creatine kinase, U L^−1^	88 (129)	88 (154)	90 (117)	0.398	0.09
Creatine kinase Isoenzyme MB, U L^−1^	12 (9)	12.6 (10.0)	11.2 (9.5)	0.097	0.15
Troponin I, ng mL^−1^	0.02 (0.09)	0.02 (0.11)	0.02 (0.05)	0.660	0.30
B‐Type natriuretic peptide, pg mL^−1^	82 (192)	82 (229)	82 (126)	0.754	0.22
Prothrombin time, s	12 (1)	12 (1)	12 (1)	0.383	0.10
Radiological abnormalities, *n* (%)					
Ground glass opacity	252 (71.8)	165 (67)	87 (82)	0.005	0.34
Pulmonary consolidation	100 (28.5)	63 (26)	37 (35)	0.080	0.20
Pulmonary interstitial abnormalities	124 (35.3)	77 (31)	47 (44)	0.020	0.27
Pneumothorax	0	0	0	‐	‐
Pleural effusion	20 (5.7)	17 (6.9)	3 (2.8)	0.127	0.19
Highest oxygen treatment level, *n* (%)				0.058	0.35
None	138 (39.3)	107 (43.7)	31 (29.2)		
Nasal cannula	139 (39.6)	94 (38.4)	45 (42.5)		
High‐Flow nasal cannula	18 (5.1)	12 (4.9)	6 (5.7)		
Non‐invasive ventilation	40 (11.4)	24 (9.8)	16 (15.1)		
Invasive ventilation	16 (4.6)	8 (3.3)	8 (7.5)		
Drug Treatment, *n* (%)					
Intravenous immunoglobin	85 (24.2)	39 (16)	46 (43)	< 0.001	0.63
Glucocorticoid	189 (53.8)	105 (43)	84 (79)	< 0.001	0.80
Paxlovid	4 (1.1)	4 (1.6)	0 (0)	0.186	0.18
Baricitinib	3 (0.9)	2 (0.8)	1 (0.9)	0.905	0.01
Antibiotics	248 (70.7)	152 (62.0)	96 (90.6)	< 0.001	0.71

BMI, body mass index; SpO_2_, pulse oxygen saturation.

^*^Comparisons are performed between the two subgroups.

### 2.2 Outcome Analysis in the Overall Cohort

**Figure 1:**
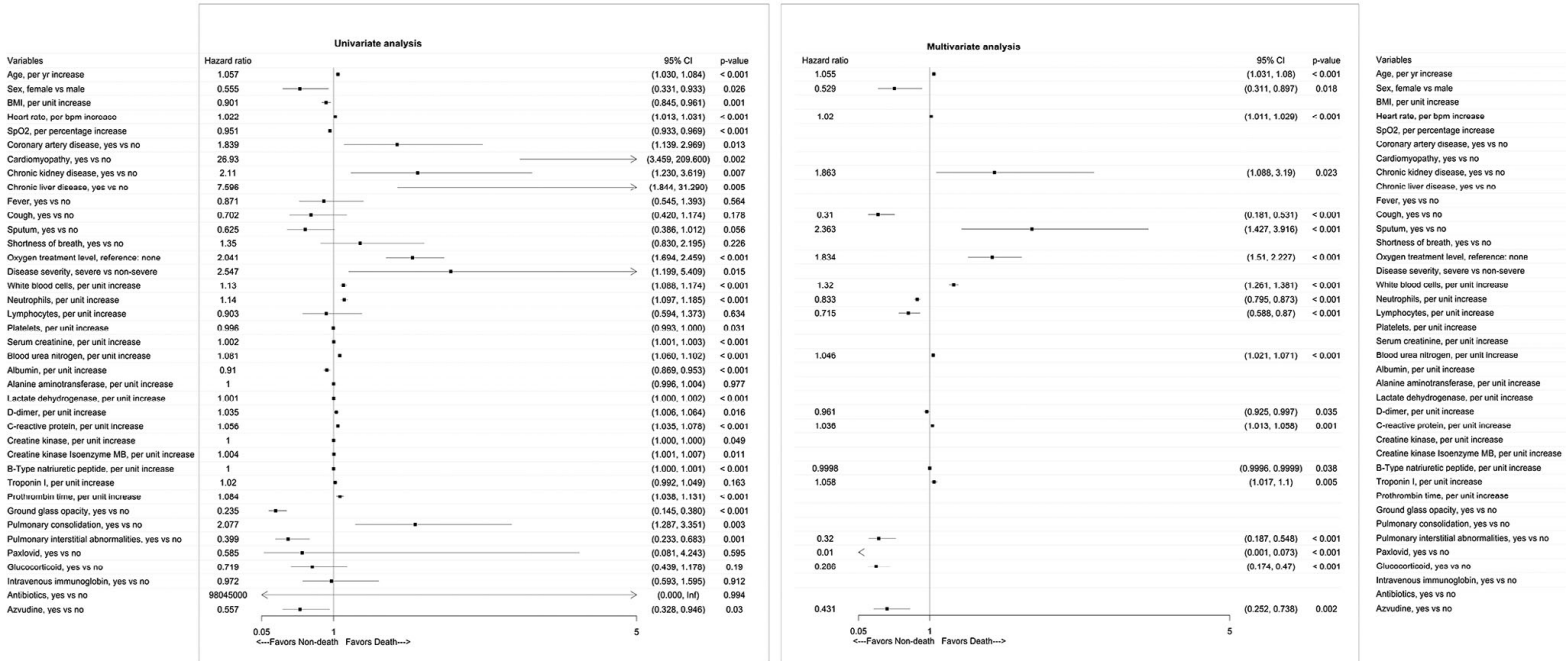
Forest plot of univariate and multivariate logistic regression analysis in the overall cohort. BMI, body mass index; SpO_2_, pulse oxygen saturation; CI, confidence interval.

**Figure 2:**
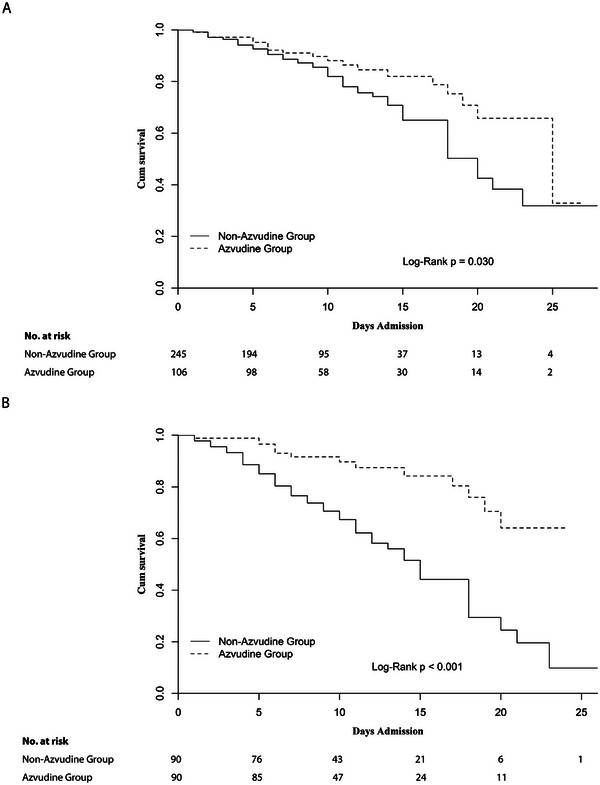
Estimated survival of the patients in the A) overall cohort and B) the propensity score‐matched cohort.

During hospitalization, 72 patients died. The predictive value for mortality of all these parameters was evaluated in the univariate COX regression analysis first (Table S1, Supporting Information). Potential predictors derived from Table 1 and univariate analysis were further adjusted in the multivariate COX regression model (**Figure** **1**). In the multivariate model, Azvudine treatment was an independent predictor for mortality (Figure 1). Patients who received Azvudine had a 56.9% lower risk of death (hazard ratio: 0.431, 95% confidence interval (CI): 0.252–0.738, *p* = 0.002) than those who did not (Figure 1). In the Kaplan–Meier analysis, patients in the Azvudine group had better in‐hospital survival than those in the non‐Azvudine group (**Figure** **2**A).

Results of the secondary outcomes are listed in Table S2 (Supporting Information). ICU admission rate and duration of ICU stay were comparable between the two groups.Acute respiratory distress syndrome (ARDS) occurred in 67 (19.1%) patients, shock in 22 (6.3%), and acute kidney injury in 22 (6.3%). Patients in the Azvudine group had lower rates of shock and acute kidney injury than those in the non‐Azvudine group. The predictive value of Azvudine therapy for shock and acute kidney injury was evaluated using univariate and multivariate logistic regression analyses (Tables S3–S6, Supporting Information). After adjustment for confounding factors, Azvudine therapy was associated with lower risks of shock (odds ratio: 0.010; 95% CI: 0.000–0.318, *p* = 0.009, Table S4, Supporting Information) and acute kidney injury (odds ratio: 0.125; 95% CI: 0.021–0.742, *p* = 0.022, Table S6, Supporting Information).

In patients who received Azvudine treatment, the median (interquartile range) time interval from disease onset and Azvudine treatment was 2.0 (2.0) days, 2.0 (2.0) days in patients who survived, and 1.0 (2.0) days in those who died (*p* = 0.141). The median (interquartile range) duration of Azvudine treatment was 6.0 (5.0) days, 13.4 (6.0) days in patients who survived, and 6.0 (3.0) days in those who died (*p* = 0.087). As for safety evaluations, there were 8 (7.5%) patients with headache, 6 (5.7%) with dizziness, and 6 (5.7%) with nausea that might be related to Azvudine. There were no serious adverse effects related to Azvudine in this study.

### 2.3 Sensitivity Analysis in the PSM Cohort

**Figure 3:**
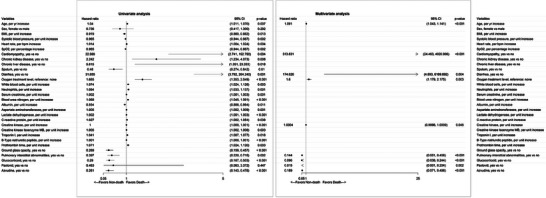
Forest plot of univariate and multivariate logistic regression analysis in the propensity score‐matched cohort. BMI, body mass index; SpO_2_, pulse oxygen saturation; CI, confidence interval.

For sensitivity analysis, an additional propensity score matching (PSM) cohort was established. The key characteristics of the PSM cohort are presented in **Table** **2** and other parameters are shown in Table S7 (Supporting Information). Univariate and multivariate COX regression analyses for predicting mortality were also performed in the PSM cohort (**Figure** **3**; Table S8, Supporting Information). After adjustment for confounding factors, Azvudine treatment was still associated with better survival (hazard ratio: 0.189, 95%CI: 0.071–0.498, *p* < 0.001). In the Kaplan–Meier analysis, patients in the Azvudine group had better in‐hospital survival than those in the non‐Azvudine group (Figure 2B).

**Table 2:** Key baseline characteristics of patients with COVID‐19 in the propensity score‐matched cohort.

	Non‐Azvudine Group (*n* = 90)	Azvudine Group (*n* = 90)	*p* value	Standardized mean difference
Age, year	75 (18)	74 (18)	0.340	0.15
Sex female, n (%)	33 (36.7)	38 (42.2)	0.446	0.11
BMI, kg m^−2^	23.7 (5.2)	23.9 (4.4)	0.387	0.10
Comorbidities, *n* (%)				
Hypertension	56 (62.2)	58 (64.4)	0.757	0.05
Coronary artery disease	40 (44.4)	39 (43.3)	0.881	0.02
Diabetes mellitus	30 (33.3)	32 (35.6)	0.754	0.05
Chronic lung disease	18 (20.0)	15 (16.7)	0.563	0.09
Chronic kidney disease	13 (14.4)	9 (10.0)	0.363	0.14
Laboratory parameters				
White blood cells, 10^9^ L^−1^	6.2 (4.0)	6.3 (3.8)	0.725	0.15
Lymphocytes, 10^9^ L^−1^	0.75 (0.59)	0.77 (0.60)	0.467	0.02
C‐reactive protein, mg L^−1^	6.1 (8.0)	5.6 (8.2)	0.642	0.10
Highest oxygen treatment level, *n* (%)			0.125	0.41
None	31 (34.4)	29 (32.2)		
Nasal cannula	25 (27.8)	40 (44.4)		
High‐Flow nasal cannula	9 (10.0)	6 (6.7)		
Non‐invasive ventilation	18 (20.0)	9 (10.0)		
Invasive ventilation	7 (7.8)	6 (6.7)		
Drug Treatment, *n* (%)				
Intravenous immunoglobin	33 (36.7)	32 (35.6)	0.877	0.02
Glucocorticoid	69 (76.7)	68 (75.6)	0.861	0.03

BMI, body mass index.

## 3 Discussion

Since there was a nationwide surge of COVID‐19 cases in China in the winter of 2022, many cardiology wards also treated patients with COVID‐19 during the pandemic. This is the first study of hospitalized patients with COVID‐19 and pre‐existing cardiovascular diseases. In this retrospective analysis, we report that Azvudine therapy was associated with a lower risk of all‐cause death, independent of demographic features, comorbidities, and other clinical characteristics.

Azvudine, initially developed for HIV treatment, is a double‐target nucleoside drug with broad‐spectrum antiviral effects against many positive‐strand RBA viruses such as HBV, HCV, and HIV.^[^ ^8^, ^13^ ^]^ Studies have shown that Azvudine was well‐tolerated and could shorten the time of nucleic acid negative conversion in patients with COVID‐19 without major drug‐related adverse events.^[^ ^8^, ^9^ ^]^ It was granted by China National Medical Products Administration to treat COVID‐19 (moderate cases from day 1 to a maximum of 14 days) in July 2022 and included in the medical reimbursement list by the National Healthcare Security Administration one month later.^[^ ^14^ ^]^ In a recent study, Azvudine showed better efficacy in reducing all‐cause death than Paxlovid in the general population.^[^ ^10^ ^]^ However, another two studies indicated that Azvudine was only associated with a lower risk of composite disease progression outcome but not death compared with Paxlovid or control in patients with pre‐existing comorbidities.^[^ ^11^, ^15^ ^]^ Our study focused on a unique cohort in which all patients had pre‐existing cardiovascular diseases (Table 1). Most patients had hypertension and coronary artery disease; some had heart failure, cardiomyopathy, valvular disease, or arrhythmia. In this unique population, the Azvudine group had a 56.9% lower risk of death than the non‐Azvudine group (Figure 1). The statistical significance remained in the PSM cohort (Figure 3). The results suggest that in patients with pre‐existing cardiovascular diseases, using Azvudine to treat COVID‐19 is reasonable.

In our study, only 1/3 of the patients used Azvudine. There were several reasons for this. First, Azyudine was only approved for moderate cases of COVID‐19 (the initial diagnosis). Second, it was not recommended for use in patients with severe liver or renal impairments. Third, during the study period, Azvudine was not included on the insurance list for certain patients with varying insurance plans.

In our study, intravenous immunoglobin was not associated with a lower risk of all‐cause mortality in both the overall and PSM cohorts after adjusting for other confounding factors (Figures 1 and 3). The result is consistent with several previous studies.^[^ ^16^, ^17^ ^]^ Unexpectedly, patients who survived had an even lower percentage of intravenous immunoglobin use than those who died. Our results suggest intravenous immunoglobin should not be routinely used in patients with COVID‐19 and pre‐existing cardiovascular diseases.

Patients with different disease severity were all included in this study. Of all patients, 202 (60.6%) were severe COVID‐19 cases. Disease severity had prognostic value in the univariate regression analysis but not in the multivariate regression analysis in the overall cohort. Interestingly, the oxygen treatment level was an independent predictor for death in both the overall cohort and the PSM cohort (Figures 1 and 3). A higher level of oxygen treatment was associated with a 1.83‐ and 1.60‐times greater risk of death in the overall cohort and the PSM cohort, respectively (Figure 1). To some extent, the oxygen treatment level represented disease severity, so there might be an interaction between the two parameters. Treating patients who need higher oxygen support with a more aggressive strategy is reasonable.

Age also had prognostic value in the present study. Patients had a median age of 74 years, and those who died were significantly older than those who survived (Table 1). In the multivariate COX regression analysis, a per year increase in age was associated with a 5.5% and 9.1% greater risk of death in the overall and PSM cohorts, respectively (Figures 1 and 3). Our data suggest that older patients with COVID‐19 and pre‐existing cardiovascular diseases might need more advanced treatment and care.

Interestingly, different radiological abnormalities seemed to have different prognostic values. Ground glass opacity was the top radiological abnormality, followed by pulmonary interstitial abnormalities and pulmonary consolidation (Table 1). Patients who died had a higher percentage of pulmonary consolidation but lower rates of ground glass opacity and pulmonary interstitial abnormalities than those who survived. In the univariate regression analysis, pulmonary consolidation was associated with a higher risk of death in the overall cohort, while ground glass opacity and pulmonary interstitial abnormalities were associated with lower risks of death in both the overall and the PSM cohorts (Figures 1 and 3). In the multivariate regression analysis, pulmonary interstitial abnormalities remained statistically significant as independent predictors for a better outcome. So pulmonary consolidation in patients with COVID‐19 should raise a red flag rather than the other two kinds of radiological abnormalities.

Many laboratory parameters had prognostic values in the univariate analysis, including inflammation factors, parameters representing liver function and kidney function, myocardial enzymes, and markers of heart failure. Among these parameters, several proved to have prognostic value in the overall cohort through multivariate analyses, while none were significant in the PSM cohort (Figures 1 and 3). It is possible that these laboratory parameters are simply indications of the severity of the disease, rather than being independent prognostic factors for patients with COVID‐19 and pre‐existing cardiovascular conditions.

As for secondary outcomes, Azvudine recipients did not have a shorter ICU stay duration than controls (Tables S2 and S9, Supporting Information). This is understandable because the duration of ICU stay was related to many factors for these patients with pre‐existing cardiovascular diseases. Azvudine therapy was not related to a lower risk of ARDS might be because patients already developed ARDS before they received Azvudine therapy. In this unique population with COVID‐19 and pre‐existing cardiovascular diseases, Azvudine therapy was associated with lower risks of shock and acute kidney injury. The findings suggested that Azvudine therapy could potentially have positive impacts on the circulation system.

### 3.1 Limitations

This study had several limitations that merit comment. First, this was a single‐center study in a tertiary hospital, which led to selection bias. But our hospital is one of the largest in Wuhan, China, which takes care of a large number of patients during the COVID‐19 pandemic. Second, this study only included patients with COVID‐19 and comorbid cardiovascular diseases hospitalized in the cardiology department, not all patients with COVID‐19. Hence the findings may not be extended to outpatients and patients without pre‐existing cardiovascular diseases. However, this is a vulnerable population, and data on Azvudine in this population is lacking. Third, this was a retrospective analysis of the real‐world experience of Azvudine. There were no interventions and other antiviral treatment groups as controls. Future multicenter randomized controlled studies comparing Azvudine and other antiviral treatments are warranted.

## 4 Conclusion

In summary, this study suggested that Azvudine therapy was associated with a lower risk of all‐cause death in hospitalized patients with COVID‐19 and pre‐existing cardiovascular diseases. Azvudine therapy was also associated with lower risks of shock and acute kidney injury in these patients.

## 5 Experimental Section

### Population

Patients with COVID‐19 admitted to the Department of Cardiology, the Central Hospital of Wuhan, Wuhan, China, between December 10, 2022, and January 10, 2023, were consecutively retrospectively enrolled in this study. The inclusion criteria were: 1) patients with confirmed COVID‐19; 2) patients with pre‐existing cardiovascular diseases, including hypertension, coronary artery disease, heart failure, cardiomyopathy, valvular heart disease, arrhythmia, and hyperlipemia. The exclusion criteria included: 1) patients younger than 18 years old; 2) patients without preexisting cardiovascular diseases. COVID‐19 was diagnosed using the reverse transcription polymerase chain reaction method. Patients with different severities of COVID‐19 were all included. According to the novel coronavirus pneumonia prevention and control program published by the National Health Committee of China (the tenth edition, Jan 2023)^[^ ^12^ ^]^ non‐severe cases were defined as those without any of the following: 1) shortness of breath, with a respiratory rate ≥30 min^−1^; 2) resting pulse oxygen saturation ≤93% (room air); 3) oxygenation index ≤300 mmHg; 4) progressively worsening symptoms, and chest imaging showing >50% increase of pulmonary lesions within 24–48 h; 5) respiratory failure and in need of mechanical ventilation; 6) shock; 7) ICU admission in the presence of failure of other organs. Severe cases were defined as patients with any one of the above conditions. The patient enrollment process is presented in Figure S1 (Supporting Information). This study was performed in accordance with the Declaration of Helsinki and was approved by the Institutional Review Board and Ethics Committee (Approval No. WHZXKYL2023‐099). Informed consent was obtained from all participants.

### Data Collection and Outcomes

Demographic features, comorbidities, vital signs, symptoms, laboratory data, chest computed tomography parameters, and treatment information were collected. The primary outcome was all‐cause death during hospitalization. Secondary outcomes included ICU admission, duration of ICU stay, ARDS, shock, and acute kidney injury.

### Statistical analysis

Continuous variables were displayed as mean ± standard deviation or median (interquartile range) of data not normally distributed. Categorical parameters were expressed as ratios or percentages. The Student *t*‐test or Mann–Whiney U test was conducted as appropriate to compare continuous data between two independent samples. The chi‐square test was used for categorical data. Univariate and multivariate COX regression analyses were conducted to identify risk factors for death. Logistic regression analyses were conducted to identify risk factors for secondary outcomes. Kaplan–Meier curve was performed, and the log‐rank *p*‐value was calculated in survival analysis. Sensitivity analysis was conducted in a cohort with similar baseline characteristics, which was derived using the PSM method. Patients with missing data (*n* = 8) were excluded (Figure S1, Supporting Information), therefore imputation was not conducted. A *p*‐value < 0.05 was considered significant. Data management and analyses were performed using R version 4.1.2.

## Conflict of Interest

The authors declare no conflict of interest.

## Author Contributions

L.W., Z.‐H.H., H.‐D.Z., and M.‐H.C. designed, studied, and performed the conception of the project. L.W., Z.‐H.H., H.‐D.Z., and M.‐H.C. analyzed and interpreted data. L.W., Z.‐H.H., H.‐D.Z., and M.‐H.C. drafted the article. L.W., Z.‐H.H., L.H., X.G., X.‐Y.L., H.‐D.Z., and M.‐H.C. performed the critical revision of the article for intellectual content. L.W., Z.‐H.H., L.H., X.G., X.‐Y.L., H.‐D.Z., and M.‐H.C. worked for final approval of the article. L.W., Z.‐H.H., and M.‐H.C. gave the provision of study materials or patients. H.‐D.Z. assisted with statistical expertise. H.‐D.Z. and M.‐H.C. acquired funds. L.W., H.‐D.Z., and M.‐H.C. provided administrative, technical, or logistic support. L.W., Z.‐H.H., L.H., X.G., and X.‐Y.L. collected data. L.W. takes responsibility for the content of the manuscript, including the data and analysis.

## Supporting information

Supporting Information

## Data Availability

Research data are not shared.
