# Supplementary material for: Azvudine for the Treatment of COVID‐19 in Pre‐Existing Cardiovascular Diseases: A Single‐Center, Real‐World Experience
Source: Adv Sci (Weinh). 2024 Mar 27;11(23):2306050. doi: 10.1002/advs.202306050 (PMC11187877; doi:10.1002/advs.202306050)

ADVANCED SCIENCE
Open Access

Supporting Information

for *Adv. Sci.*, DOI 10.1002/advs.202306050

Azvudine for the Treatment of COVID-19 in Pre-Existing Cardiovascular Diseases: A Single-Center, Real-World Experience

Liu Wu, Zhong-Han He, Ling Huang, Xin Guo, Xu-Yong Li, Hong-Da Zhang and Man-Hua Chen**

Supplementary Materials for

Azvudine for the treatment of COVID-19 in pre-existing cardiovascular diseases: a single-center, real-world experience

Liu Wu, MD, Zhong-Han He, MD, Ling Huang, MD, Xin Guo, MD, Xu-Yong Li, MD, Hong-Da Zhang, MD, PhD, Man-Hua Chen, MD.

Correspondence to: chenmh@aliyun.com and zhanghongda213@163.com

This file includes:

Tables S1 to S9 and Figure S1

Table S1. Univariate COX regression analysis for death in the overall cohort.

	**Unadjusted Hazard Ratio (95% CI)**	**p value**
**Demographic characteristics**		
Age, per yr ↑	1.057 (1.030, 1.084)	<0.001
Sex, female vs male	0.555 (0.331, 0.933)	0.026
BMI, per kg/m^2 ↑	0.901 (0.845, 0.961)	0.001
**Clinical Characteristics**		

Systolic blood pressure, per mmHg ↑	0.995 (0.983, 1.007)	0.412
Heart rate, per bpm ↑	1.022 (1.013, 1.031)	< 0.001
SpO2, per 1% ↑	0.951 (0.933, 0.969)	< 0.001
Current Smoker, yes vs no	1.659 (0.936, 2.939)	0.083
Disease Severity, severe vs non-severe	2.547 (1.199, 5.409)	0.015
**Comorbidities**		
Hypertension, yes vs no	1.143 (0.691, 1.890)	0.603
Coronary artery disease, yes vs no	1.839 (1.139. 2.969)	0.013
Heart failure, yes vs no	1.633 (0.945, 2.824)	0.079
Cardiomyopathy, yes vs no	26.930 (3.459, 209.600)	0.002
Valvular heart disease, yes vs no	0.776 (0.108, 5.601)	0.802
Arrhythmia, yes vs no	1.129 (0.578, 2.205)	0.723
Diabetes mellitus, yes vs no	1.015 (0.630, 1.634)	0.952
Chronic lung disease, yes vs no	1.118 (0.612, 2.040)	0.717
Chronic kidney disease, yes vs no	2.110 (1.230, 3.619)	0.007
Chronic liver disease, yes vs no	7.596 (1.844, 31.290)	0.005
Stroke or transient ischemic attack, yes vs no	1.790 (0.981, 3.268)	0.058
Cancer, yes vs no	2.168 (0.866, 5.431)	0.098
Immunosuppression, yes vs no	3.084 (0.424, 22.420)	0.266
**Symptoms at admission**		
Fever, yes vs no	0.871 (0.545, 1.393)	0.564
Cough, yes vs no	0.702 (0.420, 1.174)	0.178
Sputum, yes vs no	0.625 (0.386, 1.012)	0.056

Shortness of breath, yes vs no	1.350 (0.830, 2.195)	0.226
Diarrhea, yes vs no	1.494 (0.207, 10.800)	0.691
Oxygen Treatment Level	2.041 (1.694, 2.459)	< 0.001
**Laboratory parameters**		
White blood cells, per 10^9/L ↑	1.130 (1.088, 1.174)	< 0.001
Neutrophils, per 10^9/L ↑	1.140 (1.097, 1.185)	< 0.001
Lymphocytes, per 10^9/L ↑	0.903 (0.594, 1.373)	0.634
Hemoglobin, per g/L ↑	0.993 (0.983, 1.003)	0.151
Platelets, per 10^9/L ↑	0.996 (0.993, 1.000)	0.031
Serum creatinine, per umol/L ↑	1.002 (1.001, 1.003)	< 0.001
Blood urea nitrogen, per mg/dl ↑	1.081 (1.060, 1.102)	< 0.001
Alanine aminotransferase, per U/L ↑	1.000 (0.996, 1.004)	0.977
Aspartate aminotransferase, per U/L ↑	1.000 (1.000, 1.001)	0.395
Albumin, per g/L ↑	0.910 (0.869, 0.953)	< 0.001
Total bilirubin, per umol/L ↑	1.009 (0.997, 1.022)	0.129
Direct bilirubin, per umol/L ↑	1.008 (0.995, 1.021)	0.236
Lactate dehydrogenase, per U/L ↑	1.001 (1.000, 1.002)	< 0.001
D-dimer, per ug/ml ↑	1.035 (1.006, 1.064)	0.016
C-reactive protein, per mg/L ↑	1.056 (1.035, 1.078)	< 0.001
Glycosylated hemoglobin, per 1% ↑	1.012 (0.841, 1.217)	0.900
Creatine kinase, per U/L ↑	1.000 (1.000, 1.000)	0.049
Creatine kinase Isoenzyme MB, per U/L ↑	1.004 (1.001, 1.007)	0.011
Troponin elevation, per 1 unit ↑	1.020 (0.992, 1.049)	0.163

B-Type natriuretic peptide, per pg/ml ↑	1.000 (1.000, 1.001)	< 0.001
Prothrombin time, per 1s ↑	1.084 (1.038, 1.131)	< 0.001
**Radiological abnormalities**		
Ground Glass Opacity, yes vs no	0.235 (0.145, 0.380)	< 0.001
Pulmonary Consolidation, yes vs no	2.077 (1.287, 3.351)	0.003
Pulmonary Interstitial Abnormalities, yes vs no	0.399 (0.233, 0.683)	0.001
Pneumothorax, yes vs no	-	-
Pleural Effusion, yes vs no	0.950 (0.410, 2.201)	0.905
**Drug Treatment**		
Glucocorticoid, yes vs no	0.719 (0.439, 1.178)	0.190
Azvudine, yes vs no	0.557 (0.328, 0.946)	0.030
Intravenous immunoglobin, yes vs no	0.972 (0.593, 1.595)	0.912
Paxlovid	0.585 (0.081, 4.243)	0.595
Baricitinib	3.579 (0.871, 14.709)	0.077
Antibiotics	98045000 (0.000, Inf)	0.994

BMI = body mass index; SpO_2 = pulse oxygen saturation.

Table S2. Secondary outcomes in the overall cohort.

	**All patients (n = 351)**	**Non-Azvudine Group (n = 245)**	**Azvudine Group (n = 106)**	**p value[*]**
ICU Admission, n (%)	147 (41.9)	97 (39.6)	50 (47.2)	0.186
ICU Stay, days	0 (4)	0 (4)	0 (6)	0.202
ARDS, n (%)	67 (19.1)	47 (19.2)	20 (18.9)	0.945
Shock, n (%)	22 (6.3)	21 (8.6)	1 (0.9)	0.007
AKI, n (%)	22 (6.3)	20 (8.2)	2 (1.9)	0.026

ICU = intensive care unit; ARDS = acute respiratory distress syndrome; AKI = acute kidney injury.

[*] Comparisons are performed between the two subgroups.

Table S3. Univariate logistic regression analysis for shock in the overall cohort.

	Unadjusted Odds Ratio (95% CI)	p value
**Demographic characteristics**		
Age, per yr ↑	1.035 (0.993, 1.080)	0.104
Sex, female vs male	0.186 (0.054, 0.641)	0.008
BMI, per kg/m^2 ↑	0.915 (0.806, 1.040)	0.174
**Clinical Characteristics**		
Systolic blood pressure, per mmHg ↑	0.962 (0.936, 0.988)	0.005
Heart rate, per bpm ↑	1.017 (0.998, 1.035)	0.075
SpO2, per 1% ↑	0.942 (0.893, 0.993)	0.027
Current Smoker, yes vs no	0.325 (0.043, 2.483)	0.279
Disease Severity, severe vs non-severe	3.131 (1.037, 9.459)	0.043
**Comorbidities**		
Hypertension, yes vs no	1.247 (0.494, 3.143)	0.640
Coronary artery disease, yes vs no	1.213 (0.509, 2.887)	0.663
Heart failure, yes vs no	1.209 (0.342, 4.279)	0.768
Cardiomyopathy, yes vs no	0.000 (0.000, +∞)	0.999
Valvular heart disease, yes vs no	0.000 (0.000, +∞)	0.999
Arrhythmia, yes vs no	2.187 (0.764, 6.260)	0.145
Diabetes mellitus, yes vs no	1.498 (0.621, 3.615)	0.369
Chronic lung disease, yes vs no	0.861 (0.246, 3.015)	0.815
Chronic kidney disease, yes vs no	1.928 (0.617, 6.029)	0.259
Chronic liver disease, yes vs no	0.000 (0.000, +∞)	0.999

Stroke or transient ischemic attack, yes vs no	2.062 (0.658, 6.469)	0.215
Cancer, yes vs no	0.000 (0.000, +∞)	0.999
Immunosuppression, yes vs no	7.786 (0.678, 89.386)	0.099
**Symptoms at admission**		
Fever, yes vs no	0.900 (0.374, 2.165)	0.815
Cough, yes vs no	0.786 (0.320, 1.933)	0.600
Sputum, yes vs no	0.953 (0.388, 2.339)	0.916
Shortness of breath, yes vs no	0.296 (0.113, 0.774)	0.013
Diarrhea, yes vs no	0.000 (0.000, +∞)	0.999
Oxygen Treatment Level, reference: none	2.818 (1.963, 4.046)	< 0.001
**Laboratory parameters**		
White blood cells, per 10^9/L ↑	1.110 (1.016, 1.212)	0.021
Neutrophils, per 10^9/L ↑	1.118 (1.023, 1.223)	0.014
Lymphocytes, per 10^9/L ↑	0.661 (0.269, 1.623)	0.366
Hemoglobin, per g/L ↑	1.007 (0.987, 1.028)	0.475
Platelets, per 10^9/L ↑	0.989 (0.982, 0.996)	0.002
Serum creatinine, per umol/L ↑	1.002 (1.000, 1.005)	0.061
Blood urea nitrogen, per mg/dl ↑	1.071 (1.022, 1.122)	0.004
Alanine aminotransferase, per U/L ↑	1.002 (0.998, 1.006)	0.308
Aspartate aminotransferase, per U/L ↑	1.001 (1.000, 1.003)	0.115
Albumin, per g/L ↑	0.870 (0.801, 0.944)	0.001
Total bilirubin, per umol/L ↑	0.999 (0.958, 1.042)	0.964
Direct bilirubin, per umol/L ↑	1.000 (0.963, 1.038)	0.986

Lactate dehydrogenase, per U/L ↑	1.002 (1.001, 1.004)	0.002
D-dimer, per ug/ml ↑	1.031 (0.959, 1.107)	0.410
C-reactive protein, per mg/L ↑	1.053 (1.009, 1.099)	0.017
Glycosylated hemoglobin, per 1% ↑	1.319 (0.990, 1.759)	0.059
Creatine kinase, per U/L ↑	1.000 (1.000, 1.000)	0.031
Creatine kinase Isoenzyme MB, per U/L ↑	1.007 (1.001, 1.013)	0.017
Troponin elevation, per 1 unit ↑	1.038 (0.994, 1.085)	0.095
B-Type natriuretic peptide, per pg/ml ↑	1.000 (1.000, 1.001)	0.029
Prothrombin time, per 1s ↑	1.021 (0.902, 1.156)	0.745
**Radiological abnormalities**		
Ground Glass Opacity, yes vs no	0.300 (0.125, 0.718)	0.007
Pulmonary Consolidation, yes vs no	0.938 (0.356, 2.469)	0.896
Pulmonary Interstitial Abnormalities, yes vs no	1.572 (0.659, 3.749)	0.308
Pneumothorax, yes vs no	-	-
Pleural Effusion, yes vs no	0.000 (0.000, +∞)	0.998
**Drug Treatment**		
Glucocorticoid, yes vs no	1.031 (0.433, 2.452)	0.946
Azvudine, yes vs no	0.102 (0.013, 0.765)	0.026
Intravenous immunoglobin, yes vs no	2.305 (0.949, 5.599)	0.065

BMI = body mass index; SpO_2 = pulse oxygen saturation; CI = confidence interval

Table S4. Univariate and multivariate logistic regression analysis for shock in the overall cohort.

	Unadjusted<br><br>Odds Ratio (95% CI)	p-value	Adjusted<br><br>Odds Ratio
Age, per yr ↑	1.035 (0.993, 1.080)	0.104	
Sex, female vs male	0.186 (0.054, 0.641)	0.008	0.059 (0.00[illegible]
Systolic blood pressure, per mmHg ↑	0.962 (0.936, 0.988)	0.005	
SpO2, per 1% ↑	0.942 (0.893, 0.993)	0.027	
Disease Severity, severe vs non-severe	3.131 (1.037, 9.459)	0.043	
Shortness of breath, yes vs no	0.296 (0.113, 0.774)	0.013	0.084 (0.02[illegible]
Oxygen Treatment Level, reference: none	2.818 (1.963, 4.046)	< 0.001	4.180 (2.44[illegible]
White blood cells, per 10^9/L ↑	1.110 (1.016, 1.212)	0.021	
Platelets, per 10^9/L ↑	0.989 (0.982, 0.996)	0.002	
Blood urea nitrogen, per mg/dl ↑	1.071 (1.022, 1.122)	0.004	
Albumin, per g/L ↑	0.870 (0.801, 0.944)	0.001	0.859 (0.76[illegible]
Lactate dehydrogenase, per U/L ↑	1.002 (1.001, 1.004)	0.002	
C-reactive protein, per mg/L ↑	1.053 (1.009, 1.099)	0.017	
Creatine kinase Isoenzyme MB, per U/L ↑	1.007 (1.001, 1.013)	0.017	
B-Type natriuretic peptide, per pg/ml ↑	1.000 (1.000, 1.001)	0.029	
Ground Glass Opacity, yes vs no	0.300 (0.125, 0.718)	0.007	
Azvudine, yes vs no	0.102 (0.013, 0.765)	0.026	0.010 (0.00[illegible]

BMI = body mass index; SpO_2 = pulse oxygen saturation; CI = confidence interval.

Table S5. Univariate logistic regression analysis for AKI in the overall cohort.

	**Unadjusted**<br><br>**Odds Ratio (95% CI)**	**p value**

**Demographic characteristics**		
Age, per yr ↑	1.085 (1.032, 1.141)	0.001
Sex, female vs male	0.459 (0.175, 1.201)	0.113
BMI, per kg/m^2 ↑	0.756 (0.654, 0.874)	< 0.001
**Clinical Characteristics**		
Systolic blood pressure, per mmHg ↑	1.005 (0.983, 1.028)	0.662
Heart rate, per bpm ↑	1.019 (1.001, 1.038)	0.036
SpO2, per 1% ↑	0.901 (0.849, 0.957)	0.001
Current Smoker, yes vs no	1.141 (0.323, 4.029)	0.838
Disease Severity, severe vs non-severe	4.463 (1.295, 15.380)	0.018
**Comorbidities**		
Hypertension, yes vs no	1.005 (0.410, 2.464)	0.992
Coronary artery disease, yes vs no	1.474 (0.621, 3.497)	0.379
Heart failure, yes vs no	8.011 (3.202, 20.043)	< 0.001
Cardiomyopathy, yes vs no	0.000 (0.000, +∞)	0.999
Valvular heart disease, yes vs no	3.869 (0.414, 36.173)	0.235
Arrhythmia, yes vs no	1.606 (0.517, 4.984)	0.413
Diabetes mellitus, yes vs no	1.498 (0.621, 3.615)	0.369
Chronic lung disease, yes vs no	0.533 (0.121, 2.348)	0.405
Chronic kidney disease, yes vs no	3.480 (1.272, 9.524)	0.015
Chronic liver disease, yes vs no	15.619 (0.943, 258.576)	0.055
Stroke or transient ischemic attack, yes vs no	2.827 (0.976, 8.819)	0.055
Cancer, yes vs no	3.086 (0.345, 27.625)	0.314

Immunosuppression, yes vs no	0.000 (0.000, +∞)	0.999
**Symptoms at admission**		
Fever, yes vs no	0.592 (0.235, 1.491)	0.266
Cough, yes vs no	2.139 (0.706, 6.476)	0.179
Sputum, yes vs no	0.953 (0.388, 2.339)	0.916
Shortness of breath, yes vs no	1.886 (0.749, 4.746)	0.178
Diarrhea, yes vs no	0.000 (0.000, +∞)	0.999
**Oxygen Treatment Level,** reference: none	2.143 (1.545, 2.973)	< 0.001
**Laboratory parameters**		
White blood cells, per 10^9/L ↑	1.212 (1.108, 1.326)	< 0.001
Neutrophils, per 10^9/L ↑	1.226 (1.119, 1.344)	< 0.001
Lymphocytes, per 10^9/L ↑	1.031 (0.605, 1.756)	0.912
Hemoglobin, per g/L ↑	0.987 (0.969, 1.005)	0.163
Platelets, per 10^9/L ↑	0.989 (0.982, 0.996)	0.002
Serum creatinine, per umol/L ↑	1.006 (1.003, 1.010)	0.001
Blood urea nitrogen, per mg/dl ↑	1.156 (1.100, 1.214)	< 0.001
Alanine aminotransferase, per U/L ↑	1.001 (0.996, 1.006)	0.734
Aspartate aminotransferase, per U/L ↑	1.001 (0.999, 1.002)	0.438
Albumin, per g/L ↑	0.896 (0.828, 0.971)	0.007
Total bilirubin, per umol/L ↑	1.017 (0.992, 1.043)	0.181
Direct bilirubin, per umol/L ↑	1.009 (0.987, 1.032)	0.403
Lactate dehydrogenase, per U/L ↑	1.002 (1.000, 1.003)	0.014
D-dimer, per ug/ml ↑	1.075 (1.012, 1.141)	0.019
C-reactive protein, per mg/L ↑	1.061 (1.018, 1.106)	0.005

Glycosylated hemoglobin, per 1% ↑	1.319 (0.990, 1.759)	0.059
Creatine kinase, per U/L ↑	1.000 (1.000, 1.000)	0.394
Creatine kinase Isoenzyme MB, per U/L ↑	1.006 (1.000, 1.012)	0.040
Troponin elevation, per 1 unit ↑	1.029 (0.981, 1.080)	0.244
B-Type natriuretic peptide, per pg/ml ↑	1.001 (1.000, 1.001)	0.001
Prothrombin time, per 1s ↑	1.117 (1.028, 1.214)	0.009
**Radiological abnormalities**		
Ground Glass Opacity, yes vs no	0.098 (0.035, 0.273)	< 0.001
Pulmonary Consolidation, yes vs no	4.945 (2.005, 12.196)	0.001
Pulmonary Interstitial Abnormalities, yes vs no	0.846 (0.335, 2.133)	0.722
Pneumothorax, yes vs no	-	-
Pleural Effusion, yes vs no	2.898 (0.780, 10.760)	0.112
**Drug Treatment**		
Glucocorticoid, yes vs no	1.031 (0.433, 2.452)	0.946
Azvudine, yes vs no	0.216 (0.050, 0.943)	0.042
Intravenous immunoglobin, yes vs no	0.476 (0.137, 1.648)	0.241

BMI = body mass index; SpO_2 = pulse oxygen saturation; CI = confidence interval.

Table S6. Univariate and multivariate logistic regression analysis for AKI in the overall cohort.

	Unadjusted Odds Ratio (95% CI)	p-value	Adjusted Odds Ratio
Age, per yr ↑	1.085 (1.032, 1.141)	0.001	1.083

BMI, per kg/m^2 ↑	0.756 (0.654, 0.874)	< 0.001	
Heart rate, per bpm ↑	1.019 (1.001, 1.038)	0.036	
SpO2, per 1% ↑	0.901 (0.849, 0.957)	0.001	
Disease Severity, severe vs non-severe	4.463 (1.295, 15.380)	0.018	
Oxygen Treatment Level, reference: none	2.143 (1.545, 2.973)	< 0.001	1.610 (1.03[illegible]
White blood cells, per 10^9/L ↑	1.212 (1.108, 1.326)	< 0.001	1.169 (1.04[illegible]
Platelets, per 10^9/L ↑	0.989 (0.982, 0.996)	0.002	0.991 (0.98[illegible]
Serum creatinine, per umol/L ↑	1.006 (1.003, 1.010)	0.001	1.005 (1.00[illegible]
Albumin, per g/L ↑	0.896 (0.828, 0.971)	0.007	
Lactate dehydrogenase, per U/L ↑	1.002 (1.000, 1.003)	0.014	
D-dimer, per ug/ml ↑	1.075 (1.012, 1.141)	0.019	
C-reactive protein, per mg/L ↑	1.061 (1.018, 1.106)	0.005	
Creatine kinase Isoenzyme MB, per U/L ↑	1.006 (1.000, 1.012)	0.040	
B-Type natriuretic peptide, per pg/ml ↑	1.001 (1.000, 1.001)	0.001	
Prothrombin time, per 1s ↑	1.117 (1.028, 1.214)	0.009	
Ground Glass Opacity, yes vs no	0.098 (0.035, 0.273)	< 0.001	0.284 (0.08[illegible]
Pulmonary Consolidation, yes vs no	4.945 (2.005, 12.196)	0.001	
Azvudine, yes vs no	0.216 (0.050, 0.943)	0.042	0.125 (0.02[illegible]

BMI = body mass index; SpO_2 = pulse oxygen saturation; CI = confidence interval.

Table S7. Baseline Characteristics of patients with COVID-19 in the propensity score-matched cohort.

	Non-Azvudine Group (n = 90)	Azvudine Group (n = 90)	p value	Standardized mean difference
Age, yr	75 (18)	74 (18)	0.340	0.15
Sex female, n (%)	33 (36.7)	38 (42.2)	0.446	0.11
BMI, kg/m^2	23.7 (5.2)	23.9 (4.4)	0.387	0.10
Systolic blood pressure, mmHg	129 ± 19	128 ± 18	0.826	0.03
Heart rate, bpm	85 (28)	85 (21)	0.977	0.03
SpO2, %	96 (5)	97 (4)	0.272	0.11
Severe COVID-19, n (%)	73 (81.1)	67 (744.4)	0.282	0.16
Current smoker, n (%)	10 (11.1)	15 (16.7)	0.281	0.16
**Comorbidities, n (%)**				
Hypertension	56 (62.2)	58 (64.4)	0.757	0.05
Coronary artery disease	40 (44.4)	39 (43.3)	0.881	0.02
Heart failure	14 (15.6)	14 (15.6)	1.000	<0.0001
Arrhythmia	14 (15.6)	13 (14.4)	0.835	0.03
Cardiomyopathy	0 (0)	2 (2.2)	0.155	0.21
Valvular disease	1 (1.1)	1 (1.1)	1.000	<0.0001
Diabetes mellitus	30 (33.3)	32 (35.6)	0.754	0.05
Chronic lung disease	18 (20.0)	15 (16.7)	0.563	0.09
Chronic kidney disease	13 (14.4)	9 (10.0)	0.363	0.14
Chronic liver disease	2 (2.2)	0 (0)	0.155	0.21
Stroke or transient ischemic attack	12 (13.3)	10 (11.1)	0.649	0.07

Cancer	3 (3.3)	2 (2.2)	0.650	0.07
Immunosuppression	1 (1.1)	1 (1.1)	1.000	<0.0001
Other chronic diseases	7 (7.8)	11 (12.2)	0.320	0.15
**Symptoms at admission, n (%)**				
Fever	46 (51.1)	53 (58.9)	0.294	0.16
Cough	68 (75.6)	74 (82.2)	0.273	0.16
Sputum production	63 (70.0)	74 (82.2)	0.055	0.29
Shortness of breath	54 (60.0)	56 (62.2)	0.760	00.05
Diarrhea	1 (1.1)	0 (0)	0.316	0.15
**Highest oxygen treatment level, n (%)**			0.125	0.41
None	31 (34.4)	29 (32.2)		
Nasal cannula	25 (27.8)	40 (44.4)		
High-Flow nasal cannula	9 (10.0)	6 (6.7)		
Non-invasive ventilation	18 (20.0)	9 (10.0)		
Invasive ventilation	7 (7.8)	6 (6.7)		
**Laboratory parameters**				
White blood cells, 10^9/L	6.2 (4.0)	6.3 (3.8)	0.725	0.15
Neutrophils, 10^9/L	4.9 (4.2)	4.7 (3.7)	0.560	0.15
Lymphocytes, 10^9/L	0.75 (0.59)	0.77 (0.60)	0.467	0.02
Hemoglobin, g/L	123 (33)	124 (23)	0.704	0.12
Platelets, 10^9/L	174 (111)	192 (119)	0.248	0.21
Serum creatinine, umol/L	83 (78)	75 (42)	0.089	0.22
Blood urea nitrogen, mg/dl	7.8 (7.6)	6.4 (5.2)	0.042	0.29

Alanine aminotransferase, U/L	21 (20)	22 (23)	0.271	0.20
Aspartate aminotransferase, U/L	36 (32)	37 (32)	0.653	0.06
Albumin, g/L	35 (10)	34 (5)	0.426	0.10
Total bilirubin, umol/L	13 (9)	12 (10)	0.285	0.21
Direct bilirubin, umol/L	5 (4)	5 (5)	0.912	0.19
Lactate dehydrogenase, U/L	276 (171)	251 (138)	0.892	0.01
D-dimer, ug/ml	0.8 (2.1)	0.9 (1.1)	0.875	0.13
C-reactive protein, mg/L	6.1 (8.0)	5.6 (8.2)	0.642	0.10
Glycosylated hemoglobin, %	6.1 (0.8)	6.1 (0.71)	0.924	0.04
Creatine kinase, U/L	111 (220)	89 (109)	0.070	0.08
Creatine kinase Isoenzyme MB, U/L	15 (15)	10 (8)	0.003	0.17
Troponin I, ng/ml	0.03 (0.16)	0.02 (0.04)	0.032	0.35
B-Type natriuretic peptide, pg/ml	105 (501)	82 (128)	0.032	0.32
Prothrombin time, s	12 (1.6)	12 (1.3)	0.036	0.23
**Radiological abnormalities, n (%)**				
Ground glass opacity	52 (57.8)	78 (86.7)	< 0.001	0.68
Pulmonary consolidation	32 (35.6)	29 (32.2)	0.637	0.07
Pulmonary interstitial abnormalities	33 (36.7)	40 (44.4)	0.288	0.16
Pneumothorax	0	0	-	-
Pleural effusion	8 (8.9)	3 (3.3)	0.120	0.23
**Drug Treatment, n (%)**				
Intravenous immunoglobin	33 (36.7)	32 (35.6)	0.877	0.02

Glucocorticoid	69 (76.7)	68 (75.6)	0.861	0.03
Paxlovid	3 (3.3)	0 (0)	0.244	0.26
Baricitinib	2 (2.2)	1 (1.1)	1.000	0.09
Antibiotics	79 (87.8)	80 (88.9)	0.816	0.03
**Outcomes**				
Dead, n (%)	44 (48.91)	4 (15.6)	< 0.001	0.76
Hospital Stay, days	9 (8)	10 (9)	0.180	0.22
ICU Stay, days	4 (9)	0 (6)	0.028	0.32

BMI = body mass index; SpO_2 = pulse oxygen saturation.

Table S8. Univariate COX regression analysis for death in the propensity score-matched cohort.

	Unadjusted Hazard Ratio (95% CI)	p value
**Demographic characteristics**		
Age, per yr ↑	1.040 (1.011, 1.070)	0.007
Sex, female vs male	0.736 (0.417, 1.300)	0.292
BMI, per kg/m^2 ↑	0.919 (0.860, 0.982)	0.013
**Clinical Characteristics**		
Systolic blood pressure, per mmHg ↑	0.965 (0.944, 0.987)	0.002
Heart rate, per bpm ↑	1.014 (1.004, 1.024)	0.004
SpO2, per 1% ↑	0.965 (0.944, 0.987)	0.002
Current Smoker, yes vs no	1.737 (0.932, 3.240)	0.082
Disease Severity, severe vs non-severe	0.865 (0.400, 1.870)	0.712
**Comorbidities**		
Hypertension, yes vs no	1.135 (0.656, 1.965)	0.650
Coronary artery disease, yes vs no	1.376 (0.813, 2.327)	0.234
Heart failure, yes vs no	0.997 (0.532, 1.866)	0.992
Cardiomyopathy, yes vs no	22.988 (2.741, 192.790)	0.004
Valvular heart disease, yes vs no	0.887 (0.122, 6.456)	0.905
Arrhythmia, yes vs no	1.034 (0.508, 2.106)	0.926
Diabetes mellitus, yes vs no	0.992 (0.582, 1.692)	0.978
Chronic lung disease, yes vs no	1.465 (0.789, 2.721)	0.227
Chronic kidney disease, yes vs no	2.242 (1.234, 4.073)	0.008

Chronic liver disease, yes vs no	5.615 (1.351, 23.333)	0.018
Stroke or transient ischemic attack, yes vs no	1.829 (0.944, 3.542)	0.074
Cancer, yes vs no	1.618 (0.581, 4.502)	0.357
Immunosuppression, yes vs no	3.427 (0.468, 25.095)	0.225
**Symptoms at admission**		
Fever, yes vs no	0.682 (0.406, 1.147)	0.149
Cough, yes vs no	0.581 (0.315, 1.071)	0.082
Sputum, yes vs no	0.480 (0.274, 0.842)	0.010
Shortness of breath, yes vs no	1.001 (0.581, 1.726)	0.996
Diarrhea, yes vs no	31.655 (3.792, 264.240)	0.001
Oxygen Treatment Level	1.665 (1.353, 2.049)	< 0.001
**Laboratory parameters**		
White blood cells, per 10^9/L ↑	1.074 (1.024, 1.126)	0.003
Neutrophils, per 10^9/L ↑	1.084 (1.033, 1.137)	0.001
Lymphocytes, per 10^9/L ↑	0.793 (0.451, 1.393)	0.419
Hemoglobin, per g/L ↑	0.991 (0.980, 1.002)	0.116
Platelets, per 10^9/L ↑	0.997 (0.993, 1.000)	0.073
Serum creatinine, per umol/L ↑	1.002 (1.001, 1.003)	0.001
Blood urea nitrogen, per mg/dl ↑	1.068 (1.045, 1.091)	< 0.001
Alanine aminotransferase, per U/L ↑	0.997 (0.985, 1.009)	0.606
Aspartate aminotransferase, per U/L ↑	1.005 (1.002, 1.009)	0.001
Albumin, per g/L ↑	0.934 (0.886, 0.984)	0.011
Total bilirubin, per umol/L ↑	1.005 (0.991, 1.018)	0.494

Direct bilirubin, per umol/L ↑	1.009 (0.992, 1.027)	0.304
Lactate dehydrogenase, per U/L ↑	1.002 (1.001, 1.003)	< 0.001
D-dimer, per ug/ml ↑	1.017 (0.983, 1.052)	0.331
C-reactive protein, per mg/L ↑	1.027 (1.002, 1.054)	0.038
Glycosylated hemoglobin, per 1% ↑	1.007 (0.833, 1.218)	0.942
Creatine kinase, per U/L ↑	1.000 (1.000, 1.001)	< 0.001
Creatine kinase Isoenzyme MB, per U/L ↑	1.005 (1.002, 1.008)	0.003
Troponin elevation, per 1 unit ↑	1.041 (1.007, 1.077)	0.018
B-Type natriuretic peptide, per pg/ml ↑	1.001 (1.000, 1.001)	< 0.001
Prothrombin time, per 1s ↑	1.071 (1.024, 1.120)	0.003
**Radiological abnormalities**		
Ground Glass Opacity, yes vs no	0.269 (0.159, 0.457)	< 0.001
Pulmonary Consolidation, yes vs no	1.583 (0.937, 2.676)	0.086
Pulmonary Interstitial Abnormalities, yes vs no	0.397 (0.220, 0.715)	0.002
Pneumothorax, yes vs no	-	-
Pleural Effusion, yes vs no	0.605 (0.217, 1.686)	0.337
**Drug Treatment**		
Glucocorticoid, yes vs no	0.290 (0.167, 0.503)	< 0.001
Azvudine, yes vs no	0.261 (0.143, 0.478)	< 0.001
Intravenous immunoglobin, yes vs no	0.719 (0.419, 1.234)	0.231
Paxlovid	0.463 (0.063, 3.372)	0.447
Baricitinib	2.501 (0.604, 10.350)	0.206
Antibiotics	76626000(0.000, inf)	0.996

BMI = body mass index; SpO_2 = pulse oxygen saturation; CI = confidence interval.

Table S9. Outcomes in different treatment groups.

	Azvudine Group (n = 106)	Non-antiviral Group (n = 241)	Paxlovid Group (n = 4)	p value[*]
Dead, n (%)	19 (17.9)	52 (21.6)	1 (25)	0.437
Hospital Stay, days	10 (9)	7 (7)	15 (11)	< 0.001
ICU Admission, n (%)	50 (47.2)	95 (39.4)	2 (50)	0.178
ICU Stay, days	0 (6)	0 (4)	6 (20)	0.177
ARDS, n (%)	20 (18.9)	46 (19.1)	1 (25)	0.962
Shock, n (%)	1 (0.9)	20 (8.3)	1 (25)	0.008
AKI, n (%)	2 (1.9)	20 (8.3)	0 (0)	0.024

ICU = intensive care unit; ARDS = acute respiratory distress syndrome; AKI = acute kidney injury.

[*] Comparisons are performed between the first two subgroups.

Figure S1. The flow chart of patient enrollment process.

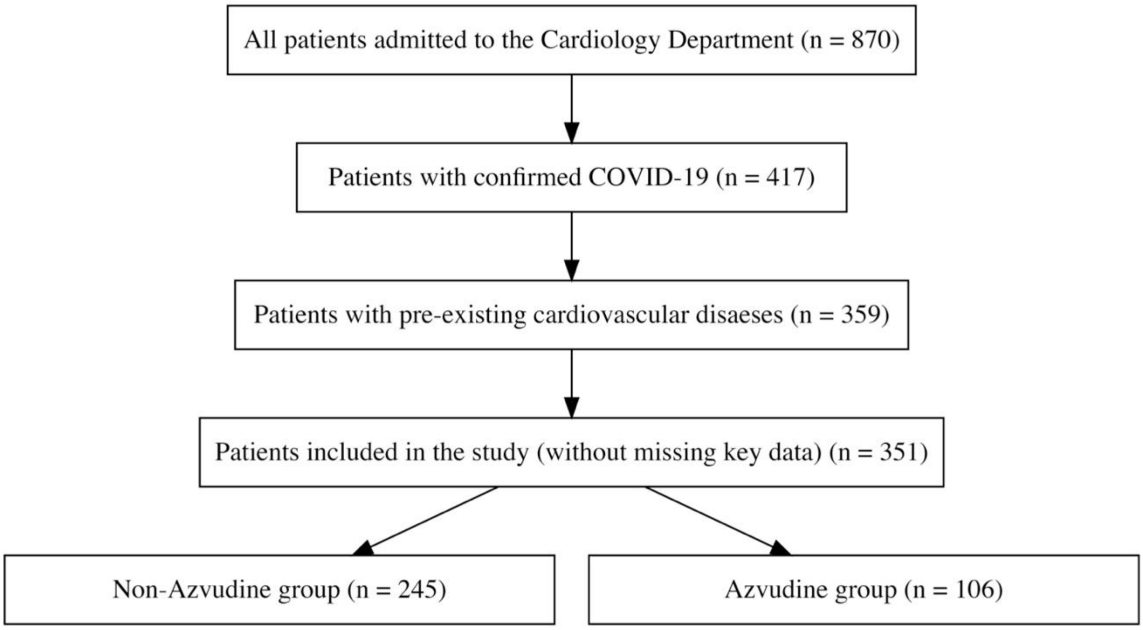

Supplement: Supplementary file 1 — Supporting Information [file ADVS-11-2306050-s001.pdf]
